# Explanatory

**Published:** 1868-07

**Authors:** 


### Editorial.

### EXPLANATORY.

We have received quite a number of enquiries in reference to the course to be pursued by the Board of Examiners under the Dental Law. Previous to the meeting of the Board we did not feel at liberty to give more than a personal opinion, and that only in a private way. We can now, after that meeting, speak with more freedom. To the question, will all be examined alike, or in just the same form? We answer: Most certainly not.

The object will be to determine the fitness for practice; and the method of ascertaining that fact would vary with different individuals.

The ability of many consists largely in knowledge based upon experience and observation, while others depend mainly upon principles, as a basis of their practical ability.

With those who have been long in practice, and the attention devoted to practical details, rather than principles, it would be unjust to press a technical examination; with most persons technicalities soon pass from the memory.

The beginner, who is fresh from the books, and the study of principles, should be examined more particularly with reference to that fact.

We are fully warranted in saying, that the Board will be inclined to leniency, so far as the welfare of the public and profession will admit; but while they will be disposed to make the examination as accommodating as practicable, yet they will remember, that justice is due to three parties in this matter, viz: the applicant, the profession, and the people.

Several enquiries have been made evidently with the impression that the Board of Examiners were in some way prosecutors for violation of this law. This is wholly a mistaken idea, the

Board has nothing more to do in that respect than any other person.

It is to the interest, and it is the duty of all persons to see that this, as well as every other good law, is not violated with impunity. We trust there may never occur an occasion for a prosecution under this law. T.

